# Fanning the flames: IFN-**γ** fuels CAR-T inflammation and cytopenia

**DOI:** 10.1172/JCI201161

**Published:** 2026-01-02

**Authors:** Stefanie R. Bailey, Marcela V. Maus

**Affiliations:** 1 Division of Pediatric Hematology/Oncology, Department of Pediatrics, Department of Microbiology, Immunology and Cancer Biology, University of Virginia, Charlottesville, Virginia, USA.; 2 Cellular Immunotherapy Program, Mass General Cancer Center, Krantz Family Center for Cancer Research, Massachusetts General Hospital, Department of Medicine, Harvard Medical School, Boston, Masschusetts, USA.

## Abstract

Chimeric antigen receptor T cell (CAR-T) therapy has transformed the treatment of hematologic malignancies, yet, severe inflammatory toxicities continue to limit its broader use. In this issue of the *JCI*, Goala et al. uncovered a mechanistic link between IFN-γ–driven inflammation and disrupted neutrophil homeostasis, revealing that cytokine release syndrome (CRS) and immune cell–associated hematologic toxicity (ICAHT) stem from a shared biological pathway. Using IL-2Ra–deficient mice and patient samples, they showed that IFN-γ suppressed IL-17A and granulocyte colony-stimulating factor (G-CSF), disrupting granulopoiesis and neutrophil survival. Strikingly, IFN-γ blockade eased both CRS and neutropenia without diminishing CAR-T efficacy, suggesting a path toward safer, better-tolerated cell therapies.

## Systemic toxicity and cytopenia arise from CAR-T therapy

CAR-T therapy has changed what is possible for patients with blood cancer, offering durable remissions in otherwise refractory disease. However, cytokine release syndrome (CRS) and immune cell–associated neurotoxicity (ICANS), which are typically managed through IL-6R or IL-1R antagonists, remain prevalent complications, especially in patients with high burdens of disease (1). In addition, prolonged cytopenias can occur, especially in patients who experienced severe CRS (2). Prolonged and severe neutropenia increases the risk for infection, extends hospital stays, and accounts for much of the nonrelapse mortality observed after CAR-T infusion (3). Collectively termed immune cell–associated hematologic toxicity (ICAHT), these cytopenias frequently appear alongside severe CRS, hinting at shared inflammatory triggers that have remained poorly defined. Prior to the publication of a study by Goala et al. (4) in this issue of the *JCI*, the biological connection between CRS and bone-marrow suppression has been largely speculative, limited by the absence of immune-competent animal models that replicate both phenomena.

## An IL-2Ra–deficient mouse model recapitulates CAR-T toxicities

Goala and colleagues fill this experimental gap by leveraging an IL-2Ra–deficient mouse model to recapitulate the dual pathology of CAR-T–mediated CRS and neutropenia (4). The choice of an IL-2Ra–deficient background was strategic, as impaired IL-2 signaling results in diminished regulatory T cell (Treg) activity (5, 6). The presence of CAR-Tregs in the infusion product has been correlated with decreased durable responses in patients with lymphoma (7), and, conversely, decreased activity of Tregs has been associated with increased toxicity (8). The murine model used by the investigators captures how unchecked cytokines amplify inflammation and simultaneously destabilize hematopoiesis. Few systems allow this level of immune complexity while retaining murine tumor immunity, making the IL-2Ra–deficient mouse model a valuable platform for dissecting CAR-T–induced toxicities in an intact host.

In the model’s immunocompetent setting, WT anti-CD19 CAR-T cells triggered high systemic levels of IL-6, TNF-a, and IFN-γ, coupled with robust M1-like macrophage polarization, resulting in a synchronized inflammatory and hematologic collapse. Following CAR-T administration, IL-2Ra–KO mice developed severe CRS accompanied by a rapid, sustained fall in circulating neutrophils. Notably, CRS occurred in the absence of tumor burden, indicating that the inflammatory response itself, and not tumor lysis, drives marrow suppression. These effects were absent in WT C57BL/6 controls, underscoring the model’s specificity for cytokine dysregulation.

## IFN-γ blockade reduces CAR-T–mediated toxicity and neutropenia

Mechanistically, excess IFN-γ skewed CD4^+^ T cell differentiation toward a Th1 phenotype while suppressing Th17-derived cytokines such as IL-17A. Because IL-17A acts upstream of G-CSF to sustain granulopoiesis (9, 10), this imbalance curtailed neutrophil production and survival. With this in mind, the authors compared clinical strategies for controlling CRS in the mouse model, specifically IL-6R and IFN-γ blockade. Both interventions dampened inflammatory cytokines without compromising antitumor efficacy, yet only IFN-γ inhibition reversed the downstream cytokine deficit — restoring IL-17A and G-CSF levels and rescuing neutrophil counts. In contrast, IL-6R blockade lowered systemic inflammation yet failed to correct cytopenia, highlighting a mechanistic distinction: IL-6 may help identify CRS, but IFN-γ drives it while simultaneously disrupting bone marrow recovery.

Upon further dissection, BrdU and Annexin V staining showed that neutrophil progenitors in IL-2Ra–deficient mice underwent less proliferation and more apoptosis, while colony-forming assays confirmed a collapse in granulocyte-monocyte progenitor potential. Both defects normalized with IFN-γ inhibition. Single-cell RNA-seq pinpointed the molecular signature: neutrophils upregulated IFN-γ–response and proapoptotic genes (*Gbp2*, *E2f1*, *Casp8*), whereas macrophages expressed *Stat1*, *Ccl5*, and *iNOS*, reinforcing an IFN-γ–dependent inflammatory circuit that links macrophage activation to neutrophil loss.

## IFN-γ/IL-17A/G-CSF axis underlies toxicity in mice and humans

Confirming this model, cotransfer of Th17 cells alongside WT CAR-T cells was sufficient to restore neutrophil proliferation and survival, increase granulocyte progenitors, and improve overall survival. Conversely, transferring Th1 cells into IFN-γ–deficient CAR-T recipients reinstated CRS and neutropenia. These elegant adoptive-transfer experiments establish IFN-γ–driven Th1 skewing as the upstream switch that connects systemic inflammation to marrow failure.

**Figure 1:**
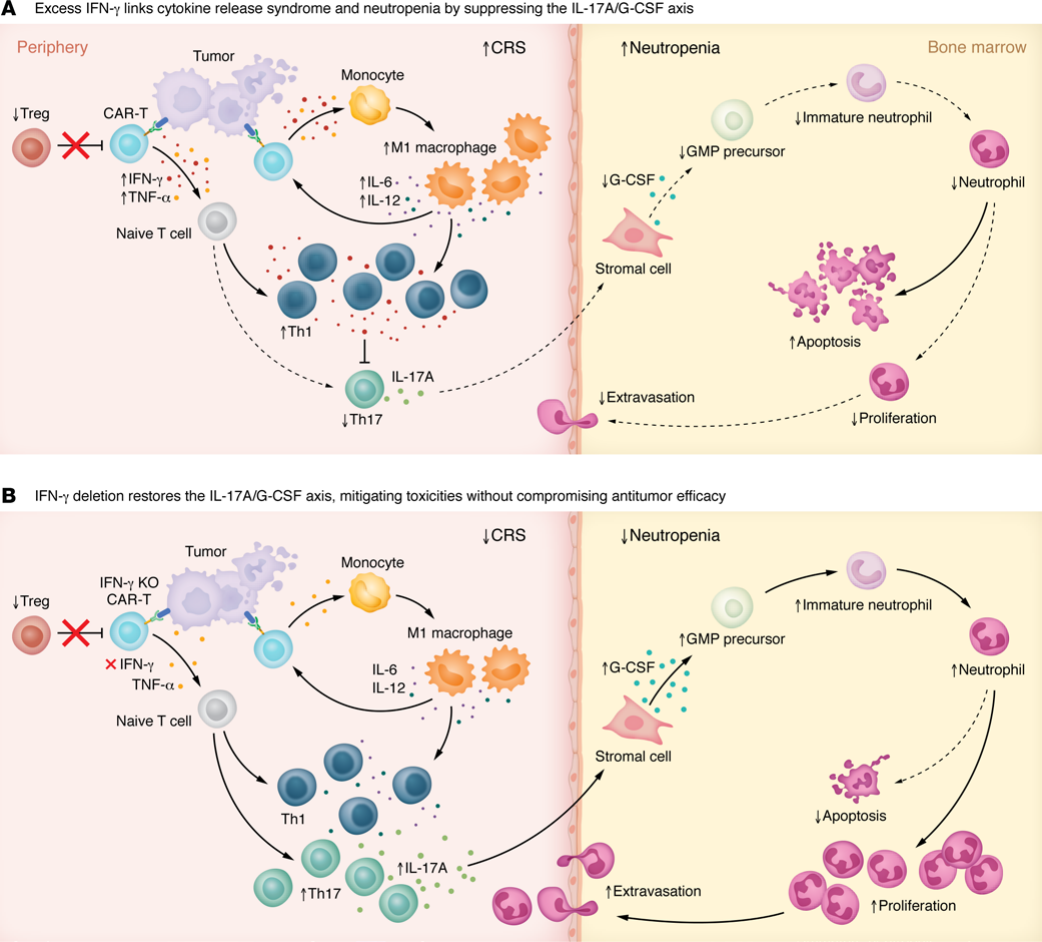
IFN-γ regulates the IL-17A/G-CSF axis to drive CRS and neutropenia. (**A**) Goala et al. (4) report that, during CAR-T therapy, IFN-γ and TNF-α released by activated CAR-T cells promoted M1-like macrophage polarization and secretion of IL-6 and IL-12, amplifying Th1 differentiation and systemic inflammation characteristic of cytokine release syndrome (CRS). Elevated IFN-γ suppressed Th17 differentiation and reduced IL-17A production, impairing IL-17A–dependent G-CSF release from bone marrow stromal cells. The resulting decrease in G-CSF diminished granulocyte progenitor proliferation, increased neutrophil apoptosis, and caused neutropenia. (**B**) Genetic deletion or blockade of IFN-γ restored Th17 differentiation and IL-17A/G-CSF signaling, normalizing granulopoiesis and mitigating neutropenia without compromising CAR-T efficacy. These findings reveal IFN-γ as a mechanistic bridge between systemic cytokine release and bone marrow suppression, suggesting that IFN-γ modulation may improve the safety of CAR-T cell therapy.

Finally, the authors sought to define the clinical relevance of these findings. Among 43 CAR-T–treated patients, those with high-grade CRS and neutropenia showed a pronounced rise in the serum IFN-γ–to–IL-17A ratio compared with patients without these toxicities. The concordance between murine and human data strengthens the biological argument and underscores the clinical relevance of the IFN-γ/IL-17A/G-CSF axis in CAR-T–driven toxicities (Figure 1).

## Conclusions and future directions

These discoveries deepen a growing recognition of IFN-γ‘s dual role in CAR-T biology. Prior studies have shown that CAR-T–derived IFN-γ activates innate immune cells, amplifying inflammation and, at times, dampening T cell persistence (11–15). Furthermore, high levels of IFN-γ in the bone marrow have been reported to associate with prolonged cytopenias (16). Goala et al. further extend this narrative, positioning IFN-γ as not merely a driver of macrophage activation but a key regulator of hematopoietic balance. By suppressing IL-17A and G-CSF, IFN-γ shifts the marrow away from granulopoiesis and toward inflammatory stress. In doing so, this study reframes CRS not as a simple cytokine surge but as a coordinated immune misalignment, spanning adaptive, innate, and myeloid compartments.

Viewed through this lens, the IL-2Ra–deficient model becomes more than a technical advance — it offers a powerful platform to interrogate how cytokine circuits intertwine across tissues and cell types, revealing strategies to mitigate inflammation without compromising therapeutic efficacy. The ability to modulate IFN-γ signaling in an immunocompetent system offers a framework for defining when and where this cytokine helps or harms.

Looking ahead, several questions emerge. How broadly does this Th1/Th17 axis operate across different CAR constructs or disease contexts? Could brief, precisely timed IFN-γ modulation during the inflammatory peak preserve efficacy while limiting toxicity? And, how can we mitigate the risk of infection or immune rebound while fine tuning this balance? Each question underscores the need to move from binary cytokine control toward interventions defined by timing, tissue, and context.

Early efforts to translate these insights are already underway. A clinical trial (NCT06550141) is now using IFN-γ–neutralizing therapy in CAR-T–treated patients, directly testing whether these mechanistic insights translate into safer outcomes. The results could redefine how the field approaches cytokine modulation — less as suppression, more as calibration.

Ultimately, Goala et al. have provided more than a new model for studying these toxicities. They have highlighted how a single cytokine can bridge inflammation and hematopoiesis, providing a conceptual framework for integrating immune regulation with cellular engineering. For hematologic malignancies, transient IFN-γ control may open the door to safer cell therapy. For solid tumors, where IFN-γ remains essential for immune clearance (17), the challenge will be to preserve its antitumor potency while avoiding collateral damage. Ultimately, the path forward lies in mastering — not muting — the power of this pivotal cytokine.

## Supplementary Material

Supplemental data

## Version 1 01/02/2026

Electronic publication
