# Supplementary material for: Fanning the flames: IFN-γ fuels CAR-T inflammation and cytopenia
Source: J Clin Invest. 2026 Jan 2;136(1):e201161. doi: 10.1172/JCI201161 (PMC12721904; doi:10.1172/JCI201161)
Supplement: Supplemental data [file jci-136-201161-s107.pdf]

Marcela V. Maus Lab/ MGH – IP Portfolio Status Report - November 19, 2025	
**Title**	**App. Num.**
METHOD AND APPLICATION FOR EX VIVO CULTURE AND STIMULATION OF T CELLS	62/444608
METHOD AND APPLICATION FOR EX VIVO CULTURE AND STIMULATION OR INHIBITION OF T CELLS	62/485679
	62/580256
METHODS AND COMPOSITIONS RELATING TO EX VIVO CULTURE AND MODULATION OF T CELLS	PCT/US2018/013215
	16/476592
	18/165174
CHIMERIC ANTIGEN RECEPTORS BASED ON ALTERNATIVE SIGNAL 1 DOMAINS	62/444605
	62/580243
	62/584060
	PCT/US2018/013213
	16/476588
	18/058707
CAR T CELL-MEDIATED SECRETION OF TOXIC DRUGS TO MODIFY THE TUMOR MICROENVIRONMENT AND ENHANCE CAR T CELL POTENCY	62/444582
	62/485670
CAR T CELL-MEDIATED SECRETION OF CYTOTOXIC DRUGS TO MODIFY THE TUMOR MICROENVIRONMENT AND ENHANCE CAR T CELL POTENCY	62/629593
CHIMERIC ANTIGEN RECEPTOR T CELLS TARGETING THE TUMOR MICROENVIRONMENT	62/658307
	PCT/US2018/027783
	2024-014208
	2019-555484
	18784599.5
	201880025361.8
	3059444
	2025205213
	2023278069
	2018251206
	62/746895
CHIMERIC ANTIGEN RECEPTORS TARGETING THE TUMOR MICROENVIRONMENT	PCT/US2019/017727
	2024-193719
	2020-542979
	19751389.8
	201980024375.2
	3090546
	2019218989
CHIMERIC ANTIGEN RECEPTOR T CELLS TARGETING THE TUMOR MICROENVIRONMENT	16/603675
	18/477532

CHIMERIC ANTIGEN RECEPTORS TARGETING THE TUMOR MICROENVIRONMENT	16/969098
TARGETED T CELLS WITH INDUCIBLE CYTOTOXICITY TOWARD IMMUNOSUPPRESSIVE CELLS	62/444595
TARGETED T CELLS WITH CYTOTOXICITY TOWARD IMMUNOSUPPRESSIVE CELLS	PCT/US2018/013095
	18738854.1
	16/475717
T CELLS EXPRESSING AN APRIL-BASED CHIMERIC ANTIGEN RECEPTOR TO TARGET BCMA OR TACI-EXPRESSING PLASMA CELLS AND ALSO AVOID ANTIGEN ESCAPE FROM ANTI-BCMA THERAPY	62/444622
T CELLS EXPRESSING AN APRIL-BASED CHIMERIC ANTIGEN RECEPTOR TO TARGET BCMA OR TACL-EXPRESSING PLASMA CELLS AND ALSO AVOID ANTIGEN ESCAPE FROM ANTI-BCMA THERAPY	62/516279
T CELLS EXPRESSING AN APRIL-BASED CHIMERIC ANTIGEN RECEPTOR	62/580258
T CELLS EXPRESSING A CHIMERIC ANTIGEN RECEPTOR	PCT/US2018/013221
	2019-536552
	18739161.0
	201880010940.5
	3048648
	2018207300
	62/629558
	62/771998
METHODS AND COMPOSITIONS FOR TREATING CANCER	62/773001
IMMUNE CELLS EXPRESSING A CHIMERIC ANTIGEN RECEPTOR	PCT/US2019/013103
	2023-104000
	2020-538037
	19738635.2
	201980017420.1
	3087476
	2019206573
T CELLS EXPRESSING A CHIMERIC ANTIGEN RECEPTOR	16/476595
IMMUNE CELLS EXPRESSING A CHIMERIC ANTIGEN RECEPTOR	16/961189
CD37-TARGETED CHIMERIC ANTIGEN RECEPTOR T CELLS FOR LYMPHOMAS/LEUKEMIAS	62/472275
CHIMERIC ANTIGEN RECEPTORS TARGETING CD37	PCT/US2018/022974
	2019-550784
	18768250.5
	18768250.5
	18768250.5

	18768250.5
	201880022534.0
	3056115
	2025202029
	2018236450
	16/493142
	17/741166
	2022-102796
	202410332918.X
ANTI-CD79B BASED CHIMERIC ANTIGEN RECEPTOR T CELLS AS TREATMENT FOR LYMPHOMA	62/516234
T CELLS EXPRESSING A CHIMERIC ANTIGEN RECEPTOR	62/627514
	PCT/US2018/036465
	2018279085
	3063169
	201880040774.3
	18812659.3
	2019-566930
	2023-089236
	2025203414
	16/620275
ANTI-CD37 CHIMERIC ANTIGEN RECEPTOR T CELLS FOR NON-HODGKIN LYMPHOMA	62/688775
CHIMERIC ANTIGEN RECEPTORS TARGETING CD37 AND CD19	62/757562
	PCT/US2019/038518
	2020-570533
	19821907.3
	201980041887.X
	3099928
	2019290230
	17/254392
COMPOSITIONS AND METHODS FOR TREATMENT OF T CELL MALIGNANCIES	62/700033
	PCT/US2019/042268
	17/260783
CHIMERIC ANTIGEN RECEPTORS TARGETING CD79B AND CD19	62/779346
	62/888795
	PCT/US2019/066357
	17/312744
ANTIBODIES AND CHIMERIC ANTIGEN RECEPTORS THAT TARGET TACI	62/856998
	62/907930

	63/012735
	PCT/US2020/036108
	2021-571870
	20818549.6
	202080054113.3
	3139057
	2020287626
	17/616274
IMMUNE EFFECTOR CELLS ENGINEERED TO BE RESISTANT TO THE IMMUNSUPPRESSIVE MICROENVIRONMENT BY INCORPORATION OF A RECEPTOR THAT TURNS THE NEGATIVE SIGNAL OF TGFBETA INTO A POSITIVE COSTIMULATORY SIGNAL	62/872323
USE OF CD70 TARGETED CHIMERIC ANTIGEN RECEPTOR (CAR) T CELLS FOR THE TREATMENT OF ACUTE MYELOID LEUKEMIA (AML)	62/900826
CD70 TARGETED CHIMERIC ANTIGEN RECEPTOR (CAR) T CELLS AND USES THEREOF	PCT/US2020/051018
	20865730.4
	17/761131
CAR-BITES: PSMA-PSCA FOR PROSTATE CANCER	62/912266
NEUTRALIZING IFN GAMMA PRODUCTION BY CAR T CELLS TO ALLEVIATE CYTOKINE RELEASE SYNDROME	62/948864
ENGINEERED IMMUNE CELLS WITH REDUCED TOXICITY AND USES THEREOF	63/005952
	20901409.1
	2020405049
	PCT/US2020/065733
	17/784935
CRISPR SCREEN IN HUMAN CAR-T CELLS	63/124022
METHODS AND COMPOSITIONS FOR IMMUNE CELL CRISPR SCREENS	63/344951
	PCT/US2023/067350
	2024-569447
	202380049362.7
	3256704
	63/464483
IFNG RECEPTOR KNOCKOUT CONFERS RESISTANCE TO CAR-T CELL KILLING IN GLIOBLASTOMA	63/124605
ANTI-MESOTHELIN ANTIBODY REAGENTS	63/275866
	202280087739.3
	PCT/US2022/079282
	2024-526723
	22891080.8

ANTI-MESOTHELIN CAR T CELLS SECRETING TEAMS AND METHODS OF USE THEREOF	63/275911
	63/278825
	202280087745.9
	2024-526840
	22891082.4
	PCT/US2022/079284
	18/707459
METHODS OF CELL ABLATION	63/297217
	PCT/US2023/060210
METHODS AND COMPOSITIONS FOR CONTROLLING T CELL ACTIVATION	63/317292
	PCT/US2023/063775
	23767584.8
	18/844805
COMPOSITIONS AND METHODS FOR REDUCING CELL THERAPY IMMUNOGENICITY	63/331773
	PCT/US2023/065784
	2024-560753
	23789180.9
	3249063
	2023254114
	18/856592
CD70 BINDING CAR-T CELLS COMPRISING CD33 BINDING T-CELL ENGAGING ANTIBODY MOLECULES	63/331758
	63/341995
	PCT/US2023/065710
	2024-560356
	23789144.5
	202380047510.1
	3248652
	2023254840
	18/856583
CHIMERIC ANTIGEN RECEPTOR-AFFINITY MOIETY (CAR-AM) CONSTRUCTS AND USES THEREOF	63/430973
CAR T MONITORING	63/488734
	63/503298
	63/559740
B-CELL MATURATION ANTIGEN (BCMA)-BINDING CAR-T CELL MONITORING	PCT/US2024/018645
OVEREXPRESSION OF BCL-2 FAMILY PROTEINS IN CAR-T CELLS AND METHODS OF USE THEREOF TREATING CANCER	63/459541
METHODS OF PRODUCING LARGE-SCALE PLASMID LIBRARIES	63/503921
	PCT/US2024/030764

CAR-T CELLS EXPRESSING VEGF BINDING PROTEINS	63/585484<br>PCT/US2024/048466
METHODS OF CENTRAL NERVOUS SYSTEM ADMINISTRATION OF COMPOSITIONS COMPRISING A CELL THERAPY AND DIMETHYL SULFOXIDE	63/587626<br>63/600569<br>PCT/US2024/049584
CHIMERIC ANTIGEN RECEPTOR (CAR) POLYNUCLEOTIDES ENCODING TRUNCATED CD19	63/590108<br>63/600552
TREATING GLIOBLASTOMA USING CAR-T CELL THERAPY	63/590141<br>63/600620<br>PCT/US2024/050967
CHIMERIC ANTIGEN RECEPTOR (CAR)-T CELL ENGAGING ANTIBODY MOLECULE (TEAM) FORMULATIONS AND PHARMACEUTICAL COMPOSITIONS	63/590128<br>63/600564<br>PCT/US2024/050985
CAR-T CELLS COMPRISING A CDKN1B GENE KNOCKOUT AND METHODS OF USE THEREOF	63/602032<br>63/717562<br>PCT/US2024/057041
MESO-FAP WITH ADAM17 INHIBITOR OR ITK INHIBITOR	63/604730<br>PCT/US2024/057896
MESOTHELIN AND MUC16 BISPECIFIC CHIMERIC ANTIGEN RECEPTOR (CAR) T CELLS	63/568354<br>PCT/US2025/020708
CAR-T CELLS COMPRISING A GLYCO-BRIDGE	63/694715
